# The Interrelationship Between Subjective Health, Cognitive Decline, and Late Life Disability: A Path Analysis

**DOI:** 10.1093/geroni/igaa057.965

**Published:** 2020-12-16

**Authors:** Sarah Hubner, Hyeon Jung Kim, Brenda Nguyen, Brooke Hansen, Julie Blaskewicz Boron

**Affiliations:** University of Nebraska Omaha, Omaha, Nebraska, United States

## Abstract

Relationships between mental, physical and cognitive health can differentially impact individuals’ ability to function in everyday life. As people age, this can further influence independence and quality of life. To better understand these relationships, the current study implemented path analysis to investigate the impact of subjective mental, physical, and cognitive health on disability. Analyses explored relationships between demographic variables, subjective mental and physical health, cognitive decline, and self-reported disability (difficulty Walking/Climbing stairs [WC], Dressing/Bathing [DB], and Doing Errands [EA]). Data from the Behavioral Risk Factor Surveillance System were examined. The most recent four waves (2015-2018) of available data from states utilizing the Cognitive Decline Module were included (50 states, two territories). Path analyses were conducted and modeled in AMOS. Measures of CFI (0.986), TLI (0.938), and RMSEA (0.046) indicate good model fit. Listwise deletion was utilized (n=212117) . Respondents were aged 45+ and were generally white (82.8%), female (58.7%), and of “good/very good” subjective general health (64.0%). Results revealed being non-white (WCΣβ=0.028, DBΣβ=0.021, EAΣβ=0.025, all p’s<.001), of older age (WCΣβ=0.124, DBΣβ=0.004, EAΣβ=0.001, all p’s<.001), female (WCΣβ=0.016, DBΣβ=0.013, EAΣβ=0.016, all p’s<.001), poorer mental health (WCΣβ=0.080, DBΣβ=0.082, EAΣβ=0.116, all p’s<.001), poorer physical health (WCΣβ=0.410, DBΣβ=0.294, EAΣβ=0.314, all p’s<.001), and presence of subjective cognitive decline (WCΣβ=0.107, DBΣβ=0.107, EAΣβ=0.138, all p’s<.001) all had a positive total effect on disability. Ultimately, these results indicate the interrelationship between subjective health and self-reported ability/disability. These findings may help to improve care considerations for an aging population by serving as indicators for needs for assistance and support.

